# Instant rule-out of suspected non-ST-segment elevation myocardial infarction using high-sensitivity cardiac troponin T with Copeptin versus a single low high-sensitivity cardiac troponin T: findings from a large pooled individual data analysis on 10,329 patients

**DOI:** 10.1007/s00392-020-01712-y

**Published:** 2020-07-15

**Authors:** Evangelos Giannitsis, K. Huber, C. W. Hamm, M. Möckel

**Affiliations:** 1 Department of Cardiology, Angiology and Pulmology, Medizinische Klinik III, University Hospital of Heidelberg, Im Neuenheimer Feld 410, 69120 Heidelberg, Germany; 2 3rd Department of Internal Medicine, Cardiology and Intensive Care Medicine, Wilhelminen Hospital, Vienna, Austria; 3 Medical School, Sigmund Freud University, Vienna, Austria; 4 Department of Cardiology, Kerckhoff Heart and Thorax Centre, Bad Nauheim, Germany; 5 Department of Cardiology, University Hospital Giessen and Marburg, Giessen, Germany; 6 Department of Emergency Medicine, Campus Mitte and Virchow, Berlin, Germany; 7 Department of Cardiology, Charité-Universitätsmedizin Berlin, Berlin, Germany

**Keywords:** Early diagnosis, Outcomes, Effectiveness, Copeptin, High sensitivity troponin T

## Abstract

**Background:**

Evidence is sparse and inconsistent on the role of a dual marker strategy (DMS) combining Copeptin with cardiac troponin T (cTnT) for instant rule-out of a non-ST-segment myocardial infarction (NSTEMI) when high sensitivity cardiac troponin T (hs-cTnT) is used.

**Methods:**

Data on 10,329 patients from 5 trials were pooled to evaluate initial Copeptin in combination with hs-cTnT against a single marker strategy (SMS) based on hs-cTnT < limit of detection. Endpoints were sensitivities and negative predictive values (NPV) for rule-out of NSTEMI, 30-day all-cause mortality and rates of applicability for DMS or SMS.

**Results:**

NPV for rule-out of NSTEMI was high, exceeding 99.0% for the lower limits of the 95% confidence intervals (99.0% vs 99.2%) for DMS and SMS, and NPV for all cause death at 30 days was similar with very low mortality after rule-out [0.07% (0.0–0.4%) vs 0.0% (0.0–1.2%), *p* = 1.0], but applicability was 2.4-fold higher [64.6% (63.0–66.2%) vs 27.9% (26.2%—29.7%), *p* < 0.001] with DMS than SMS. In a secondary analysis on DMS after inclusion of high risk patients, performance and applicability were similar.

**Conclusion:**

Findings corroborate the 2015 European Society of Cardiology recommendation to use dual marker strategy for instant rule-out of NSTEMI, extending evidence to hs-cTnT. Novel data demonstrate a comparably safe and effective instant rule-out with Copeptin in combination with hs-cTnT versus a single marker strategy based on very low hs-cTnT but a more than twofold higher applicability of the dual marker strategy without the need to exclude very early presenters or other important subgroups.

**Graphic abstract:**

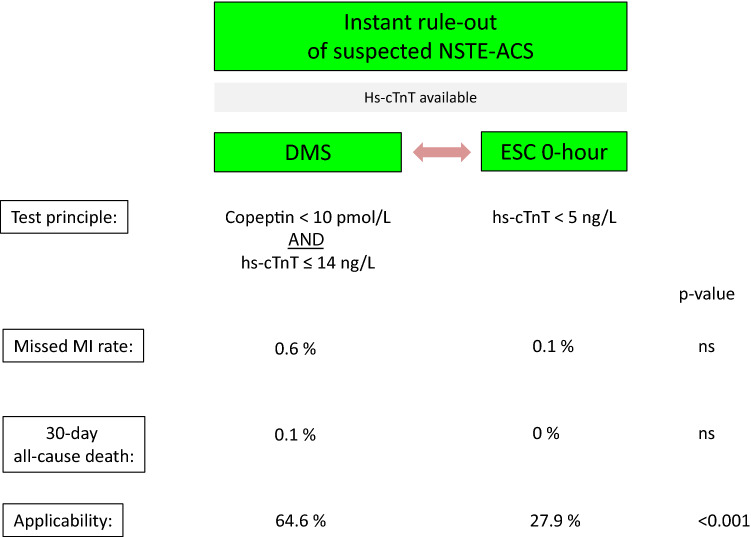
 Dual marker strategy using hs-cTnT at 99th percentile and Copeptin versus ESC 0-h immediate rule-out based on hs-cTnT < limit of detection

The use of high-sensitivity cardiac troponin (hs-cTn) assays has enabled acceleration of the diagnostic process of acute myocardial infarction (MI) in patients presenting with suspected non-ST-segment elevation acute coronary syndrome (NSTE-ACS). The various options recommended by current ESC guidelines [1] include serial hs-cTn measurements with a re-testing within 1–3 h, an option to rapidly rule out an MI based on a single measurement of hs-cTn, or alternatively, the use of a dual-marker strategy (DMS). Both strategies are appealing as they allow to exclude an MI without a second blood draw, leading to a reduced length of ED stay, and facilitating safe discharge in low-risk patients [2]. The DMS concept uses the combination of a normal cTn or hs-cTn value below the 99th percentile of a healthy reference population together with a normal Copeptin below the 95th upper limit of normal (10 pmol/L). The likelihood for an evolving or recent MI is highly unlikely when both biomarkers are normal at presentation due to the distinct reverse release kinetics of Copeptin and cTn [2].

Both strategies have shortcomings that have to be balanced against their advantages. In particular, the single hs-cTn-based strategy (SMS) requires an appropriate interval of 2–3 h between onset of symptoms and blood sampling to avoid under-diagnosis of MI due to the “troponin blind interval”. In addition, the single low hs-cTn strategy can only be applied for a small proportion (10–25%) of eligible candidates for rule-out [1]. Furthermore, this strategy strictly requires the use of an approved hs-cTn assay. In contrast, the DMS has been validated for a wide range of cTn assays including hs-cTn, contemporary or conventional sensitive cTn assays including point-of-care (POCT) tests [2, 3].

Despite ample evidence in the literature, the usefulness of DMS has recently been challenged by the increasing use of hs-cTn assays and faster protocols, measures that are assumed to diminish the benefits of DMS [4].

Therefore, we performed an analysis pooling individual data of 10,329 patients with suspected NSTE-ACS from 5 studies and evaluated the diagnostic and prognostic performance of DMS against the standard serial testing algorithm, irrespective of the hs assay sensitivity, and also compared against the competing SMS at the LoD cutoff (data not shown) (Table 1).

**Table 1:** Overview on study cohorts and study information

Variable	Full population	BIC-8 [2]	CHOPIN [6]	ProCore [3]	Diagnostic evaluations^a^	Study from Wilhelminen Hospital^a^
Population (*N*, %)	10,329 (100.0%)	888 (100.0%)	1927 (100.0%)	2279 (100.0%)	4078 (100.0%)	1157 (100.0%)
hs-cTn (*N*, %)	4597 (44.5%)	881 (99.2%)	0 (0.0%)	1478 (64.9%)	2238 (54.9%)	0 (0.0%)
NSTEMI (*N*, %)	976 (9.4%)	11 (1.2%)	116 (6.0%)	77 (3.4%)	619 (15.2%)	153 (13.2%)
30-day all-cause death (*N*, %)	33 (0.3%)	2 (0.2%)	10 (0.5%)	15 (0.7%)	0 (0.0%)	6 (0.5%)
30-day all-cause death missing (*N*, %)	4095 (39.6%)	14 (1.6%)	0 (0.0%)	0 (0.0%)	4078 (100.0%)	3 (0.3%)
Sex male (*N*, %)	5905 (57.2%)	563 (63.4%)	1082 (56.1%)	1299 (57.0%)	2290 (56.2%)	671 (58.0%)
Sex male missing (*N*, %)	15 (0.1%)	0 (0.0%)	0 (0.0%)	0 (0.0%)	15 (0.4%)	0 (0.0%)
GRACE < 109 (*N*, %)	6442 (62.4%)	738 (83.1%)	1454 (75.5%)	1410 (61.9%)	2049 (50.2%)	791 (68.4%)
GRACE 109–140 (*N*, %)	2307 (22.3%)	123 (13.9%)	374 (19.4%)	437 (19.2%)	1128 (27.7%)	245 (21.2%)
GRACE > 140 (*N*, %)	1310 (12.7%)	13 (1.5%)	99 (5.1%)	176 (7.7%)	901 (22.1%)	121 (10.5%)
GRACE missing (*N*, %)	270 (2.6%)	14 (1.6%)	0 (0.0%)	256 (11.2%)	0 (0.0%)	0 (0.0%)
Time since symptom onset < 3 h (*N*, %)	3855 (37.3%)	239 (26.9%)	1449 (75.2%)	551 (24.2%)	1257 (30.8%)	359 (31.0%)
Time since symptom onset > 3 h (*N*, %)	5604 (54.3%)	496 (55.9%)	463 (24.0%)	1556 (68.2%)	2362 (57.9%)	727 (62.8%)
Time since symptom onset missing (*N*, %)	870 (8.4%)	153 (17.2%)	15 (0.8%)	172 (7.5%)	459 (11.3%)	71 (6.1%)
Age (mean, SD) (*N*, %)	60.6 (17.5)	54.1 (15.6)	56.3 (12.8)	57.8 (17.6)	65.2 (18.6)	61.4 (16.8)
Age missing (*N*, %)	0 (0.0%)	0 (0.0%)	0 (0.0%)	0 (0.0%)	0 (0.0%)	0 (0.0%)
eGFR (mean, SD) (*N*, %)	90.1 (21.9)	96.9 (16.6)	87.1 (21.2)	93 (21.7)	-	84.7 (24.6)
eGFR missing (*N*, %)	4265 (41.3%)	101 (11.4%)	42 (2.2%)	21 (0.9%)	4078 (100.0%)	23 (2.0%)
Diabetes mellitus (*N*, %)	2123 (20.6%)	121 (13.6%)	554 (28.7%)	344 (15.1%)	909 (22.3%)	195 (16.9%)
Diabetes mellitus missing (*N*, %)	290 (2.8%)	0 (0.0%)	8 (0.4%)	0 (0.0%)	222 (5.4%)	60 (5.2%)
Smoker (*N*, %)	3117 (30.2%)	289 (32.5%)	635 (33.0%)	622 (27.3%)	1187 (29.1%)	384 (33.2%)
Smoker missing (*N*, %)	701 (6.8%)	23 (2.6%)	1 (0.1%)	0 (0.0%)	511 (12.5%)	166 (14.3%)
CAD history (*N*, %)	3491 (33.8%)	229 (25.8%)	726 (37.7%)	654 (28.7%)	1511 (37.1%)	371 (32.1%)
CAD history missing (*N*, %)	343 (3.3%)	18 (2.0%)	39 (2.0%)	49 (2.2%)	211 (5.2%)	26 (2.2%)
CAD family history (*N*, %)	2369 (22.9%)	224 (25.2%)	666 (34.6%)	472 (20.7%)	844 (20.7%)	163 (14.1%)
CAD family history missing (*N*, %)	3058 (29.6%)	61 (6.9%)	449 (23.3%)	822 (36.1%)	1219 (29.9%)	507 (43.8%)
Hypertension (*N*, %)	6645 (64.3%)	513 (57.8%)	1344 (69.7%)	1180 (51.8%)	2885 (70.7%)	723 (62.5%)
Hypertension missing (*N*, %)	327 (3.2%)	11 (1.2%)	11 (0.6%)	84 (3.7%)	184 (4.5%)	37 (3.2%)
Hypercholesterolemia (*N*, %)	4518 (43.7%)	386 (43.5%)	1042 (54.1%)	702 (30.8%)	1971 (48.3%)	417 (36.0%)
Hypercholesterolemia missing (*N*, %)	741 (7.2%)	28 (3.2%)	70 (3.6%)	182 (8.0%)	333 (8.2%)	128 (11.1%)
Recruitment period	September 2009 – March 2017	April 2011–May 2013	September 2009–October 2010	June 2015–April 2017	2009–2010	February 2011–March 2017
Follow-up time specified in protocol	NA	30 days	180 days	30 days	No follow-up	NA

*NA* not available

^a^Studies were not published

In the entire cohort, NPV and sensitivities were significantly higher (all *p* < 0.001) when Copeptin was added to either a non-hs-cTn or a hs-cTn, and findings were consistent across subgroups of special interest (all *p* for interaction NS) including early presenters (< 3 h after onset of symptoms), GRACE score risk categories, age, sex, renal function, or history of CAD (data on file).

For the present sub-analysis, the analysis was restricted to cases where the 5th gen. hs-cTnT (Roche Diagnostics) was used, and only for DMS (*n* = 3487) against SMS (*n* = 2540). For the DMS strategy, patients with a GRACE score > 140 were excluded as were patients presenting within 3 h after onset of symptoms for the SMS. The two strategies were compared regarding their performance to rule out an NSTEMI, their prediction of 30-day all-cause death, and for their applicability, i.e. the proportion of patients eligible for the respective strategy. The findings are summarized in Fig. 1. The distribution of time from onset of symptoms to ED admission is displayed in Fig. 2.

**Fig. 1:**
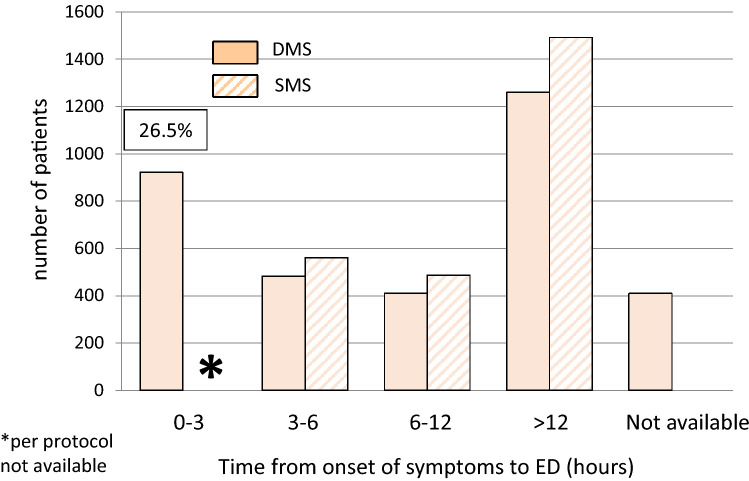
Comparison of DMS (blue) vs SMS (red) regarding effectiveness (**a**), i.e. percent eligible candidates for the respective strategy (with corresponding 95% confidence intervals). The number of eligible patients qualifying for the respective strategy is 2.3-fold higher with DMS (*p* < 0.001). NPVs for rule-out of NSTEMI (**b**) and NPVs for 30-day all-cause mortality (**c**) are shown on the right-hand side for DMS and SMS. NPVs for rule-out of NSTEMI [99.4% (99.0–99.6) vs 99.9% (99.2–100), *p* = 0.21] and prediction of 30-day all-cause death [99.9% (99.6–100) vs 100.0% (98.8–100), *p* = 1.0] were similar for DMS (blue dot for point estimate and blue lines for 95% confidence interval) versus SMS (corresponding red dots and lines)

**Fig. 2:**
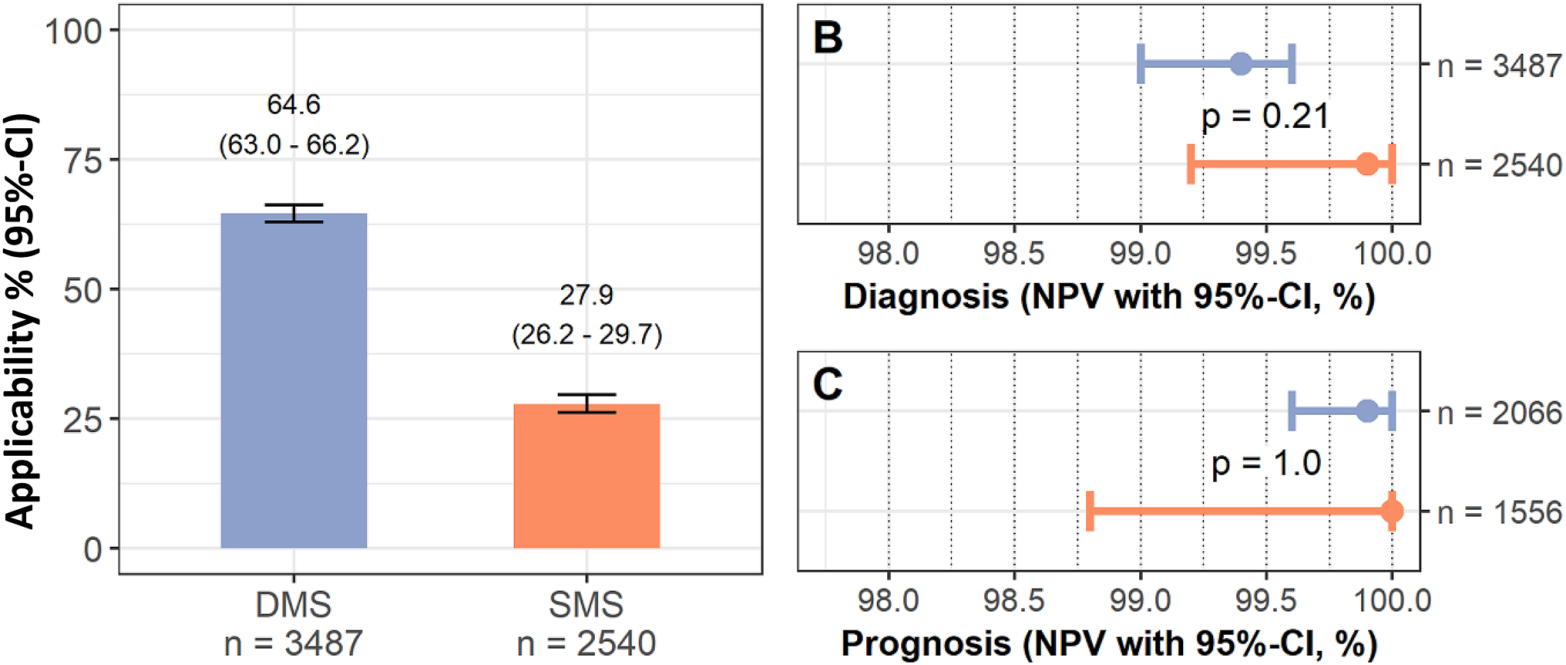
Distribution of intervals between onset of symptoms and ED admission. The distribution of intervals between symptom onset and admission to ED is very similar for time intervals between 3 and 12 h. Per protocol (2015 ESC guideline recommendation) patients with onset of symptoms 3 h or less before presentation were not eligible for the SMS strategy

The results demonstrate a comparable efficient rule-out of NSTEMI for DMS vs SMS (*p* = NS) with high confidence (lower limits of 95% confidence intervals: 99.0% vs 99.2%, comparably low rates of all-cause death at 30 days after rule-out (0.07% vs 0%) with lower confidence bound for DMS > 99.5% (99.6 vs 98.8% lower CI).

The most striking difference was the 2.3-fold higher applicability of DMS versus the single hs-cTnT strategy [64.6% (63.0–66.2%) vs 27.9% (26.2–29.7%), *p* < 0.001].

Given that hs-cTn assays allow a more accurate identification of small infarcts and demonstrate a superior risk stratification at the 99th percentile value, we repeated the analysis on diagnostic and prognostic performance as well as applicability after the inclusion of patients with a GRACE score > 140 points. We found a similar diagnostic performance of DMS versus SMS regarding negative predictive values [99.4% (95% CI 98.9–99.6) vs 99.8% (95% CI 99.2–99.9)] and sensitivities [96.2% (95%CI 93.8–97.7) vs 99.6% (95% CI 97.8–99.9)] to rule out an NSTEMI, and a consistently higher applicability [58.7% (95% CI 57.1–60.2) vs 27.9% (95% CI 26.2–29.7)] of DMS versus SMS.

Likewise, negative predictive values of DMS vs SMS were similar for prediction of death at 30 days [99% (95% CI 99.6–99.9) vs 100% (95% CI 98.8–100)].

Thus, the diagnostic and prognostic performance of DMS remained high despite inclusion of high-risk patients, while rates of suitable candidates for the algorithm (applicability) slightly declined as only a fraction of the patients at high risk are anticipated to present with normal values of hs-cTnT and Copeptin. As shown in Fig. 2, the DMS strategy included 923 of 3,487 patients (26.5%) presenting within 3 h after onset of symptoms. These early presenters are not eligible for the SMS strategy at all.

These data extend available information on the usefulness of DMS combined with hs-cTn, particularly for the Roche 5th gen. hs-cTnT assay at the 99th percentile cutoff. In addition, accumulating evidence from the Biomarker-in-Cardiology 8 (BIC-8) trial, a large randomized intervention trial [2] backed by a multicenter registry [3], supports the diagnostic value in real world, as well as the safety of discharge after instant rule-out. Additional strengths include evidence on cost savings from an economic analysis of BIC-8 trial [5], and the flexibility to perform DMS with either a non-hs-cTn (including point-of care) or an hs-cTn assay, depending on laboratory infrastructure or setting.

A potential disadvantage, that hitherto has limited the faster adoption of DMS, is the need to run a non-fully automated analyzer [2, 3]. Nevertheless, the KRYPTOR laboratory analyzer has the capability to provide Copeptin results within 10 min.
